# An Investigation of the Influence of Tyrosine Local Interactions on Electron Hopping in a Model Protein

**DOI:** 10.3390/molecules29020350

**Published:** 2024-01-10

**Authors:** Curtis A. Gibbs, Nikta Ghazi, Jody Tao, Jeffrey J. Warren

**Affiliations:** Department of Chemistry, Simon Fraser University, 8888 University Drive, Burnaby, BC V5A 1S6, Canada

**Keywords:** electron transfer, hopping, tyrosine, azurin, protein microenvironments, reduction potential tuning

## Abstract

Multi-step electron transfer reactions are important to the function of many cellular systems. The ways in which such systems have evolved to direct electrons along specific pathways are largely understood, but less so are the ways in which the reduction–oxidation potentials of individual redox sites are controlled. We prepared a series of three new artificial variants of *Pseudomonas aeruginosa* azurin where a tyrosine (Tyr109) is situated between the native Cu ion and a Ru(II) photosensitizer tethered to a histidine (His107). Arginine, glutamine, or methionine were introduced as position 122, which is near to Tyr109. We investigated the rate of Cu^I^ oxidation by a flash-quench generated Ru(III) oxidant over pH values from 5 to 9. While the identity of the residue at position 122 affects some of the physical properties of Tyr109, the rates of Cu^I^ oxidation are only weakly dependent on the identity of the residue at 122. The results highlight that more work is still needed to understand how non-covalent interactions of redox active groups are affected in redox proteins.

## 1. Introduction

**Figure 1:**
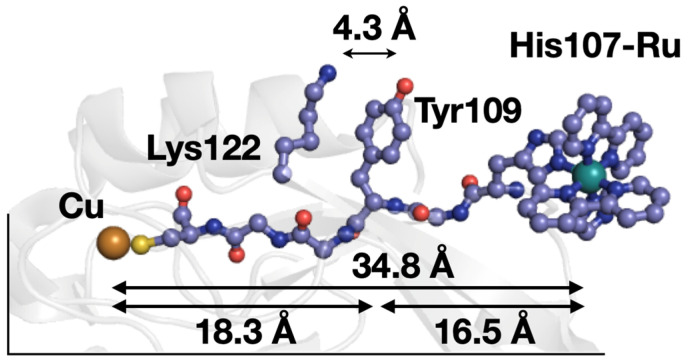
PyMOL-generated model showing the relative positions of the azurin Cu site, Tyr109, and His107-Ru(2,2’-bipyridyl)_2_imidazole. The coordinates were generated from the known Ru-labeled coordinates in Reference [19] (PDB ID 4HHG). The black lines indicate where the protein structure is truncated. The double headed arrows show through-bond distances between key groups.

Reduction–oxidation reactions of tyrosine (Tyr) are necessary components of a range of redox proteins, such as photosystem II [1], ribonucleotide reductase [2], and cyclooxygenase enzymes [3]. Even the redox reactivity of aromatic amino acids in well-investigated proteins, such as cytochrome *c* peroxidase, continue to fascinate researchers [4,5,6,7]. Furthermore, Tyr residues have been proposed to be part of protective redox chains of amino acid residues in metalloproteins [8,9,10]. Most of these examples employ long-range (≥20 Å) electron transfer (ET), highlighting the importance of Tyr in long-range redox reactions. Such ET reactions often involve ≥2 individual ET steps, which is a process called hopping [8,11,12,13]. While many functional hopping systems that employ Tyr are known in biological systems (e.g., those noted above), efforts to describe and understand the features fundamental to their function are ongoing. Here, we use three variants of *Pseudomonas aeruginosa* azurin to investigate how local interactions of Tyr affect a two-step hopping reaction (Figure 1).

Mechanistic studies of the redox chemistry of Tyr in proteins and in models show that it predominantly reacts via proton-coupled electron transfer (PCET). While Tyr radical cations have been detected using transient techniques [14,15,16], redox reactions of Tyr commonly involve the loss (or gain) of 1 proton and 1 electron (1H^+^ + 1e^−^). Tyr oxidation to the corresponding neutral radical (Tyr•) typically occurs via one of two mechanisms: concerted transfer of H• from Tyr to a suitable acceptor or sequential proton loss (generating the anionic tyrosinate, Tyr^−^) followed by oxidation of Tyr^−^ to Tyr•. In water, the latter mechanism is common [17,18]. As such, the rate of electron hopping can, in principle, be related to the rate at which Tyr is deprotonated to Tyr^−^. This reaction depends on factors such as the solution composition (pH, buffer, ionic strength) and the surrounding protein environment. The azurin variants described here are designed to probe the latter effect on Tyr-mediated hopping.

Protein model systems that probe features of Tyr-mediated hopping have been described. The peptide-based models from Giese use Tyr as the electron donor and probe the importance of different intermediate hopping sites situated between Tyr and an acceptor [20,21,22]. A range of *E. coli* artificial ribonucleotide reductase enzymes have been produced as mechanistic probes, where redox active Tyr are replaced with unnatural amino acid analogs [23,24,25,26]. There also are two other known azurin-based systems, analogous to those described here. The first used nitrotyrosinate (NO_2_Tyr^−^) to investigate designed hopping systems that employ pure ET (i.e., no PCET was required) [19]. Interestingly, the NO_2_Tyr109 system showed distinct behavior where the measured p*K*_a_ values was small with respect to the related model NO_2_Tyr122 (i.e., p*K*_a_ values of 6.0 ± 0.05 and 7.2 ± 0.05, respectively). Likewise, the formal potential (*E*°′) for NO_2_Tyr109(•/−) was larger than that of NO_2_Tyr122(•/−) (i.e., 1.2 V and 1.1 V, respectively). An X-ray diffraction structure shows that Lys122 interacts with NO_2_Tyr109, which was used to explain its unusual thermochemical properties. Specifically, the cationic Lys was proposed to stabilize the anionic NO_2_Tyr109, lowering its p*K*_a_ and raising its reduction potential. The second related system used unmodified Tyr [27]. Tyr109 again showed p*K*_a_ properties that were the same within experimental error as those for Tyr122 (10.2 ± 0.3 and 10.5 ± 0.1, respectively), and the formal potentials are not reported. The presence of Lys122 was used to explain the differences in the properties of the Tyr109 and Ty122 hopping models. Herein, we systematically vary the identity of the amino acid residue at position 122 to probe how local interactions affect Tyr-mediated hopping.

## 2. Results and Discussion

Three new azurin variants were produced based on the known “all Phe” azurin scaffold where all of the naturally occurring Tyr and Trp are replaced with Phe (i.e., Trp48Phe, Tyr72Phe, and Tyr108Phe), and additional modifications in this work include His83Gln, Gln107His, and Met109Tyr. The residue at position 122 (Lys in the wild-type protein) was substituted with Glu, Gln, Met, and Arg using standard site-directed mutagenesis protocols. Unfortunately, the Lys122Glu variant, designed to place an anionic group near Tyr109, had very low expression yields and further study was not possible. The proteins were characterized by UV-visible spectroscopy, fluorescence spectroscopy, and mass spectrometry (Figures S1–S6). Full experimental details are available in the Supporting Information. The characteristic charge transfer band of azurin (628 nm) was unchanged by the amino acid substitutions. Likewise, the π–π* band (ca. 280 nm) of Tyr109 is not affected by the identity of the amino acid residue at position 122.

**Figure 2:**
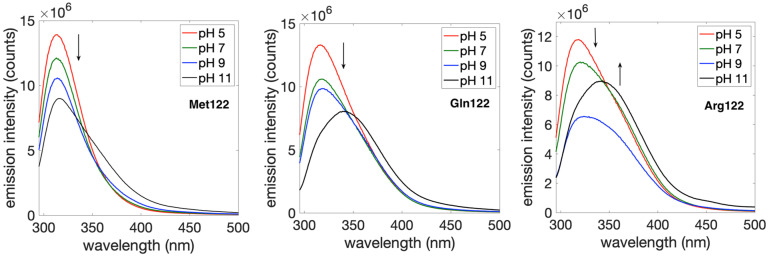
Fluorescence spectra of 60 µM solutions of His107Tyr109(Met/Gln/Arg)122 azurins in 50 mM sodium phosphate buffer at the given pH values. The excitation wavelength was 280 nm. All samples were deoxygenated, and data were collected under a N_2_ atmosphere. The arrows indicate intensity changes as pH is increased. A figure showing spectra at all pH values is given in the Supporting Information (Figure S2).

The fluorescence properties of Tyr are sensitive to pH and can also be influenced by local interactions in the protein. The p*K*_a_ of the Tyr phenolic proton is 10.3 in the ground state and about 4 in the excited state [28]. The free amino acid residue, Tyr, emits at 302 nm in the protonated (phenol) form and at 345 nm for the deprotonated (phenolate) form. We probed the pH dependent emission properties of Tyr109 in each azurin variant between pH 4 and 11 in 50 mM sodium phosphate. The emission maxima are set out in Table 1 and the corresponding spectra are shown in Figure 2 and in the Supporting Information Herein, we cannot assess the degree of proton transfer from the excited state [28]. At pH 4, the presence of Arg122 red shifts the Tyr109 emission by about 4 nm with respect to Met122. For all of the variants, the emission spectra have intensity that depends on pH and generally show decreases in intensity as the pH is increased. For the Gln122 and Arg122 variants, there are small shifts in the peak maxima until pH 11, where the emission maximum shifts to ca. 340 nm, suggesting that Tyr109 is likely deprotonated, at least partially. The spectra for the Met122 variant show a much weaker shift in emission maximum at high pH. In general, the peak shapes are indicative of different mixtures of tyrosine and tyrosinate [29]. The data suggest that the p*K*_a_ value of Tyr109 is higher in the Met122 variant (with respect to the other variants). However, the stability of azurin at pH > 10 is questionable, so precise determination of p*K*_a_ values was not possible. Likewise, emission from tyrosinate is usually weaker than for tyrosine [29]. Thus, the increase in emission intensity at pH 11 for the Arg122 variant could be an indication of changes to the protein structure at this high abiological pH. A definitive conclusion is beyond the scope of this work.

**Figure 3:**
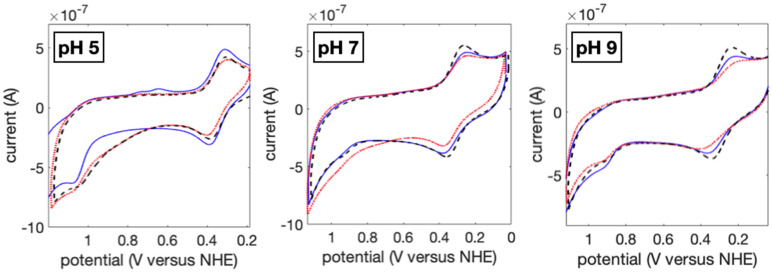
Examples of cyclic voltammograms 60 µM His107Tyr109(Met/Gln/Arg)122 azurins at pH 5, 7, and 9 in 100 mM NaOAc solutions. The blue solid line shows the Met122 variant, the red dotted line shows the Gln122 variant, and the black dashed line shows the Arg122 variant.

The Tyr reduction potentials were investigated using three voltammetry techniques: cyclic voltammetry (CV), differential pulse voltammetry (DPV), and square wave voltammetry (SWV). Additional data are provided in Figures S7–S9. In all experiments, the characteristic Cu(II/I) wave near 0.3 V versus NHE is observed. The CV experiments (Figure 3) show clear reversibility of this wave, as is expected. Likewise, the Cu(II/I) potentials shift with pH, as has been reported [30,31]. For oxidation of Tyr109, a shoulder is apparent in the CV traces (see Supporting Information), but the wave shows only a weak signal on return, suggesting irreversibility. The DPV waves showed similar intensity in the Cu- and Tyr-based redox events at lower pH values, which can indicate a degree of reversibility. The peak potentials in Table 2 are assigned from the DPV experiment. SWV experiments have been used to generate Pourbaix diagrams for reversible redox reactions of Tyr in model proteins [25,32,33], and we also carried out such experiments. The return wave in the SWV traces for Tyr is weak (see Supporting Information), suggesting irreversibility under the experimental conditions used. Thus, the reported peak potentials for Tyr109 could have error associated with the overall oxidation and degradation reactions. Ultimately, all of the Tyr potentials are within experimental error, in contrast to observations for Tyr fluorescence where the Met122 variant showed more distinct behavior.

The three azurins were modified with Ru(II)(2,2′-bipyridyl)_2_imidazole at amino acid residue His107 following procedures found in the literature [34,35,36,37]. Full experimental details are provided in the Supporting Information and the UV-vis spectra of the Ru-modified proteins shown in Figure S6. We note that we used the solution conditions (250 mM imidazole + 100 mM NaCl) from Reference [27] for kinetics experiments. Those conditions were shown to minimally affect ET kinetics for time resolved kinetics experiments (in contrast to lower buffer concentrations). Unfortunately, these conditions could not be used for fluorescence and electrochemistry experiments due to interference from the high imidazole concentration. Solution composition is an important consideration in studying PCET reactions (e.g., references [17,18,38]) and further work is needed to benchmark how it affects the redox properties of exposed amino acid residues in proteins.

**Figure 4:**
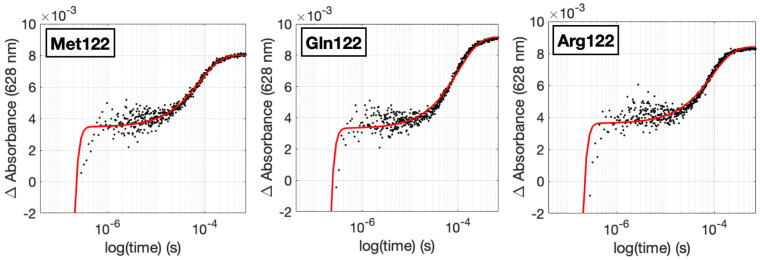
Typical time-resolved laser spectroscopy kinetics traces at 628 nm showing Cu^I^ oxidation by flash-quench-generated Ru(III) oxidants. Data are in black dots and fits to a first order kinetics model are in red. Additional kinetics traces are provided in the Supporting Information (Figures S10–S16).

The modified proteins were characterized by mass spectrometry and UV-visible spectroscopy, both of which showed successful Ru attachment at His107. The rates of Cu(I) oxidation were determined using time-resolved laser spectroscopy to monitor the reaction of flash-quench-generated Ru(III) oxidants, as previously described [19,27,34,37,39]. Briefly, the irradiation of the Ru(II) photosensitizer affords an electronically excited state (*Ru(II)) that is oxidized by an added Ru(NH_3_)_6_^3+^ electron acceptor to yield the protein-tethered Ru(III) oxidant. Because *Ru(II) is emissive, a bleach in absorbance is observed at short times in the kinetics traces for Cu(I) oxidation as shown in Figure 4 (additional traces are in the Supporting Information, Figures S10–S16). A reaction sequence showing all steps is provided in the Supporting Information (Figure S17). The rate constants for Cu(I) oxidation are set out in Table 3.

The rate constants for Cu^I^ oxidation (Table 3) are uniformly larger than reported for single-step ET (2.4 ± 0.5 10^2^ s^−1^) [34,35], which is consistent with a hopping mechanism. This was previously shown to be the case for His107Tyr109Lys122 azurin [27]. Specifically, the most probable mechanism for Try-mediated hopping was proposed to proceed via deprotonation of Tyr to the corresponding tyrosinate, followed by oxidation by Ru(III). The reaction is completed by Cu(I) oxidation by the Tyr radical. Given the single amino acid variations used here and the close similarity in observed rate constants, it is unlikely that the mechanism is different.

One surprising observation is that there are only small differences in the *k*_obs_ values at a given pH. The low pH values have somewhat higher associated errors since the rate of Cu(I) oxidation is proposed to be dependent on the rate of deprotonation of Tyr [27]. The one possible exception are the values at pH 9, where the variant with Met122 has about a 40% larger rate constant than the known Lys122 variant. Given the similarity of the observed rate constants, it is unlikely that the local Tyr109-Lys122 interaction plays the only role in changing the physical properties of Tyr109 as they relate to hopping. We note that other small deviations in the fits of the slowest rate constants may also be due to a small degree of recombination between the flash-quench generated Ru(III) label and Ru(II)(NH_3_)_6_, though such reactions are slow (0.1 to 1 ms) under our conditions [27]. Our control experiments using a model complex (Ru(bpy)_2_(imidazole)_2_(PF_6_)_2_) show recombination kinetics occur at timescales longer than 0.5 ms.

**Figure 5:**
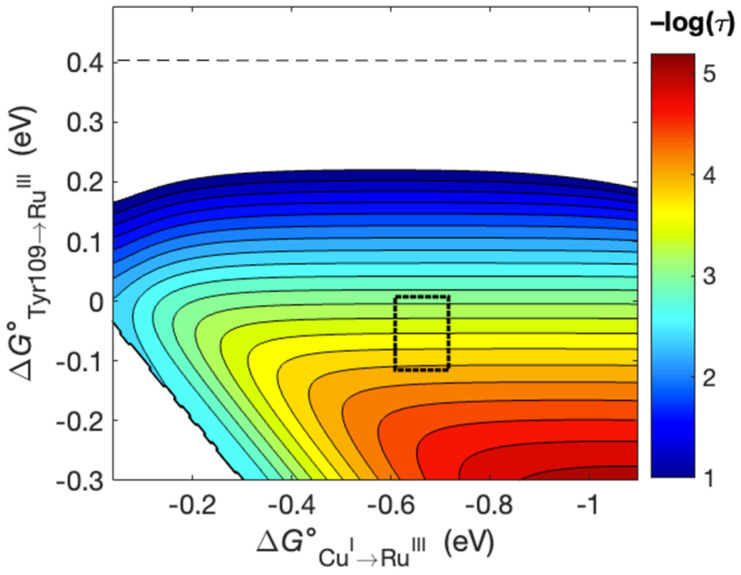
Hopping map for RuHis107Tyr109X122 azurins. The map was generated using through-bond distances where r(Ru-Tyr109) = 16.5 Å, r(Tyr109-Cu) = 18.3 Å, and r(Ru-Cu) = 34.8 Å. The λ value was 0.8 eV, T = 298K, and the distance decay constant β = 1.1 Å^−1^. The dashed line at the top shows the driving force for ET from Tyr109 to Ru(III) assuming a potential of 1.4 V for Tyr109^•+/0^. The dashed rectangle indicates the driving force regime relevant here.

The observed *k*_obs_ values (Table 3) can be analyzed using semi-classical ET theory as formulated in hopping maps [40,41,42]. A hopping map for the His107Tyr109X122 azurins described here can be constructed from the known coordinates of the known His107-NO_2_Tyr109 derivative (Figure 1, PDB ID 4HHG) [19]. The map is shown in Figure 5. Using the reduction potentials in Table 2, the reported potential for Ru(II)(2,2′-bipyridyl)_2_(imidazole)_2_^3+/2+^ (1.0 V versus NHE, [43]), and a generic hopping system with the metric set out in Figure 5, the calculated ET rate constants are 2.6 × 10^3^ s^−1^ (log *k* = 3.41) for reactions at pH 6 and 8.9 × 10^3^ s^−1^ (log *k* = 3.95) for reactions at pH 9. An alternative hopping map is set out in the Supporting Information (Figure S18). In general, the calculated rate constants are in agreement with experiment within the usual limitations of semi-classical ET theory (i.e., Marcus theory) [9,44]. The change in rate constants (a factor of about 1.2) is well-predicted with this hopping analysis.

The independence of the ET rate on the identity of the residue at position 122 is surprising given the discrepancy in the properties of Tyr109 and Tyr122 in related model proteins [19,27]. In particular, the behavior of the Met122 variant described here seems to be at odds with our other findings, i.e., the small increase in *k*_obs_ at high pH contrasts with the insensitivity of the fluorescence to pH. We do note that the observed reduction potentials for the Met122 variant depend on pH at about the same degree as the Arg122 and Gln122 variants. In another protein model that we investigated, Met and Leu were placed near Trp in a better-defined protein pocket. There, a shift in +30 mV for the Trp^•+/0^ was observed for the Leu variant versus the Met variant [45]. Although that was not a hopping system, it does illustrate the Met can influence the potentials of redox-active amino acid residues.

Overall, the changes that the residue at position 122 exert on the physical properties of Tyr109 are small. For example, the largest change in formal Tyr reduction potentials in these azurin variants is 0.02 V. Using predictions from semiclassical ET theory, this corresponds to about a 1.5-fold change in *k*_ET._ However, it is these small changes that can affect the viability of functional redox systems. Improved model systems would place the redox-active site in a better-defined protein pocket where the environment can be systematically varied. Work from Tommos using artificial peptide-based systems is one excellent example of this idea [32,46,47]. In the end, connections must continue to be built between artificial and natural protein models.

## 3. Materials and Methods

Full experimental details and additional data are provided in the Supporting Information. All reagents and materials were obtained from Sigma-Aldrich and not purified further unless noted. Luria-Bertani (LB) broth miller was purchased from BioShop Canada and prepared according to the manufacturer. Water used was from a Barnstead EASYpure system (18 MΩ cm^−1^). Q5 DNA polymerase and DpnI enzyme were purchased from New England Biolabs (NEB)UV-Visible spectrophotometry was carried out using a Cary 100-Bio spectrophotometer. MALDI mass spectrometry was carried out on a Bruker microflex LT MALDI Biotyper mass spectrometer. All laser irradiation was carried out using a home-built spectrometer containing a Continuum Surelite SLI-10 (Nd:YAG) laser, a Continuum Surelite OPO, a 75 W Xe arc lamp, and a home-built detection system.

A plasmid containing Phe mutations for all Trp and Tyr residues within azurin was a gift from Harry B. Gray and John H. Richards (California Institute of Technology, Pasadena, CA, USA). The Phe109Tyr mutation was added followed by Lys122Arg/Met/Glu/Gln so that four distinct protein scaffolds could be expressed (His107Tyr109Arg122, His107Tyr109Met122, His107Tyr109Glu122, and His107Tyr109Gln122). All mutations were introduced using site-directed mutagenic PCR using standard protocols [48]. Sanger sequencing to confirm the mutations was carried out by Eurofins Operon. All azurin variants were expressed and purified using standard literature protocols (see Supporting Information). Protein purities and concentrations were determined spectrophotometrically using reported extinction coefficients (ε(628 nm) = 5600 ^−1^ cm^−1^) [39,49]. Ruthenium-modified azurins were prepared following protocols found in the literature. Concentrations of each Ru-modified azurin was calculated spectrophotometrically using reported extinction coefficients (ε(628 nm) = 5600 M^−1^ cm^−1^ for Cu(II) and ε(500 nm) = 10,000 M^−1^ cm^−1^ for Ru(II)).

Fluorescence experiments were carried out using a Horiba Jobin-Yvon Fluorolog spectrophotometer. Protein Samples (65 μM) were prepared in 50 mM NaPi at pH values from 4 to 11. Samples were placed into air-free cuvettes and deoxygenated with 15–20 pump-backfill cycles using N_2_. Excitation was achieved using 280 nm light and spectra were recorded from 295 to 500 nm to monitor for Tyr and/or TyrO^−^ fluorescence. Ten scans were performed per sample and emission maxima were determined by averaging each series.

Electrochemical experiments were carried out using a CH Instruments 6171B potentiostat. A standard three-electrode setup was used containing a basal-plane graphite (BPG) working electrode, a platinum wire counter electrode, and an AgCl/Ag reference electrode with all samples being reported versus NHE. Protein samples (100 μM) were prepared in 100 mM NaOAc at pH values ranging from 5 to 9. The BPG electrode surface were prepared by thoroughly washing with deionized H_2_O, lightly coating with MicroPolish Powder (CH Instruments), and polishing using an microfiber polishing pad. CV spectra were collected using a 0.02, 0.04, and 0.1 V/s scan rate; however, the 0.02 V/s data are the only scans shown here unless otherwise noted. The scan window for DPV was -0.1 to 1 V at 0.003 V increments with a 0.05 V amplitude. The pulse and sample width were 0.1 and 0.02 s, respectively. The pulse period was 0.5 s.

All laser experiments were carried out with a 480 nm excitation with 6 mJ/pulse energy. Samples were reduced using L-ascorbic acid and then desalted into 250 mM imidazole and 100 mM NaCl at various pH values (5–9) for a final concentration of 35 μM. Sample volumes were 1.5 mL and special air-free cuvettes were custom made by the SFU Glass Shop to allow for deoxygenation. Then, 15-20 pump-backfill cycles using N_2_ were performed to remove O_2_ from the sample and cuvette. Prior to TA experiments, time-dependent fluorescence scans were performed, monitoring at 670 nm. The quencher [Ru(NH_3_)_6_]Cl_3_, dissolved in 250 mM imidazole, 100 mM NaCl at the required pH, was added to a final concentration of 10 mM to each sample as an exogenous quencher. A total of 15–20 pump-backfill cycles using N_2_ were performed again to remove O_2_ from the sample and cuvette. Fluorescence experiments were repeated to ensure fluorescence quenching. TA was then carried out, monitoring at 490 nm pr 628 nm for Ru(II) reduction and Cu(I) oxidation, respectively. Data were plotted and analyzed using MATLAB and MATLAB’s Curve Fitting Toolbox. Rate constants were determined using a two-exponential fit. The first exponential function describes signal from residual fluorescence due to excited state decay (and quenching) of the Ru photosensitizer and the second function describes the Cu(I) oxidation event. For the 490 nm (Ru traces), the first function is for the excited state decay of *Ru(II) and the second function is for Ru(III).

## 4. Conclusions

The results described in this report underscore that there is still much to learn about how protein environments tune the properties of redox cofactors. However, we are not the first to note anomalous behavior of hopping systems where there is a Met intermediate between the donor and the acceptor [50]. Consistent with that study, the reduction potential for the Ru(III) oxidant is insufficient of oxidize Met122, which is proposed to have a very high potential (ca. 1.6 V) [51,52]. We note that this present analysis does not consider protein dynamics [53,54,55]. While the ET pathway is not likely to change based on the identity of the residue at position 122, changes in pH can affect the ionization state of surface residues which in turn may influence the solution dynamics of the Ru label and/or Tyr109 [56,57]. While much is understood about tyrosine redox chemistry in models [18,32,58,59,60,61], more work is needed to understand how local interactions and protein dynamics affect the redox properties of amino acids and metal sites in proteins.

## Figures and Tables

**Table 1:** Tyr emission maxima of His107Tyr109(Met/Gln/Arg)122 ^a^.

pH =	4	5	6	7	8	9	10	11
**His107Tyr109Met122**	313	313	314	313	314	314	314	315
**His107Tyr109Gln122**	316	316	316	317	317	318	319	340
**His107Tyr109Arg122**	317	317	318	319	321	321	324	341

^a^ Deoxygenated samples of 65 μM azurin in 50 mM sodium phosphate buffer. Error on all values is estimated to be ±1 nm.

**Table 2:** Cu(II/I) and Tyr109(•/0) peak potentials in His107Tyr109(Met/Gln/Arg)122 azurins ^a^.

pH =	5	6	7	8	9
**Cu**	0.348	0.339	0.312	0.292	0.288
**Tyr109Met122**	1.04	0.975	0.930	0.877	0.876
**Tyr109Gln122**	1.02	0.962	0.927	0.898	0.893
**Tyr109Arg122**	1.02	0.980	0.912	0.880	0.876

^a^ Potentials reported in V versus NHE. Estimated errors are ±10 mV.

**Table 3:** Rate constants (*k*_obs_) for Cu^I^ oxidation by flash-quench generated Ru(III) oxidants ^a^.

pH	Met122	Gln122	Arg122	Lys122 ^b^
**6**	(6.8 ± 0.5) × 10^3^ 3.83	(6.3 ± 0.6) × 10^3^ 3.80	(5.4 ± 1.1) × 10^3^ 3.73	(5.7 ± 0.3) × 10^3^ 3.76
**7**	(1.2 ± 0.1) × 10^4^ 4.09	(1.1 ± 0.1) × 10^4^ 4.03	(1.1 ± 0.1) × 10^4^ 4.05	(1.2 ± 0.2) × 10^4^ 4.08
**8**	(1.8 ± 0.1) × 10^4^ 4.26	(1.6 ± 0.1) × 10^4^ 4.19	(1.6 ± 0.1) × 10^4^ 4.19	(1.7 ± 0.2) × 10^4^ 4.23
**9**	(2.6 ± 0.1) × 10^4^ 4.42	(2.1 ± 0.1) × 10^4^ 4.33	(2.2 ± 0.1) × 10^4^ 4.32	(1.8 ± 0.2) × 10^4^ 4.26

^a^ Rate constants in s^−1^. The second value in each column is log*k*_obs_. Ru(III) = Ru(III)(2,2′-bipyridyl)(imidazole)(His107). ^b^ Data from reference [27].

## Data Availability

All data are available on request.

## Supplementary Materials

The following supporting information can be downloaded at: https://www.mdpi.com/article/10.3390/molecules29020350/s1, Full experimental details. Figure S1: UV-vis spectra or azurins; Figure S2: Tyr fluorescence spectra at additional pH values; Figures S3–S5: MALDI mass-sec of azurins; Figure S6: UV-vis spectra of Ru-modified azurins; Figure S7: Cyclic voltammetry data at additional pH values; Figure S8: Differential pulse voltammetry data at additional pH values; Figure S9: Square wave voltammetry data at selected pH values; Figures S10–S16: additional kinetics data at different pH values; Figure S17: Schematic of an electron hopping mechanism Figure S18: Additional hopping maps.
