# Supplementary material for: An Investigation of the Influence of Tyrosine Local Interactions on Electron Hopping in a Model Protein
Source: Molecules. 2024 Jan 10;29(2):350. doi: 10.3390/molecules29020350 (PMC10818705; doi:10.3390/molecules29020350)
Supplement: Supplementary file 1 [file molecules-29-00350-s001.zip › molecules-2802106-supplementary.pdf]

Supporting Information for:

An Investigation of the Influence of Tyrosine Local Interactions on Electron Hopping in a Model Protein

Curtis A. Gibbs, Nikta Ghazi, Jody Tao, and Jeffrey J. Warren*

Simon Fraser University
Department of Chemistry
8888 University Drive
Burnaby BC V5A1S6 Canada
**jjwarren@sfu.ca*

Contents:

1.Experimental Details S2
2. Site-directed mutagenesis S2
3. Protein Expressions, Purification, and Modification S3
4. Tyrosine Fluorescence S3
5. Electrochemistry S4
6. Transient Absorption Spectroscopy S4
7. UV-visible spectra for His107Tyr109X122 azurins S5
8. Fluorescence spectra for X122 azurins S6
9. Mass spectrometry data for His107Tyr109X122 azurins S7
10. UV-visible spectra Ru-modified His107Tyr109X122 azurins S8
11. Cyclic voltammetry data for His107Tyr109X122 azurins S9
12. Differential Pulse voltammetry data for His107Tyr109X122 azurins S10
13. Example Square Wave voltammetry data for His107Tyr109X122 azurins S11
14. Transient Absorbance Traces for His107Tyr109X122 azurins S12
15. Additional hopping maps S18
16. References S19

1. Experimental Details

All reagents and materials were obtained from Sigma-Aldrich and not purified further unless noted. Luria-Bertani (LB) broth miller was purchased from BioShop Canada and prepared according to the manufacturer. Water used was from a Barnstead EASYpure system (18 MΩ cm-1). UV-Visible spectrophotometry was carried out using a Cary 100-Bio spectrophotometer. MALDI mass spectrometry was carried out on a Bruker microflex LT MALDI Biotyper mass spectrometer. All laser irradiation was carried out using a home-built spectrometer containing a Continuum Surelite SLI-10 (Nd:YAG) laser, a Continuum Surelite OPO, a 75 W Xe arc lamp, and a home-built detection system.

2. Site-Directed Mutagenesis

A plasmid containing Phe mutations for all Trp and Tyr residues within azurin was a gift from Harry B. Gray and John H. Richards (California Institute of Technology). The Phe109Tyr mutation was added followed by Lys122Arg/Met/Glu/Gln so that four distinct protein scaffolds could be expressed (His107Tyr109Arg122, His107Tyr109Met122, His107Tyr109Glu122, and His107Tyr109Gln122). All mutations were introduced using site-directed mutagenic PCR using standard protocols.[1] DNA primers were purchased from Eurofins Genomics. Q5 DNA polymerase and DpnI enzyme were purchased from New England Biolabs (NEB). PCR products were transformed into competent DH5α E. coli cells obtained from NEB and selected using ampicillin-containing agar plates. Colonies were grown overnight at 37 °C and single colonies selected for liquid cultures in LB broth containing 100 µg/mL of ampicillin. Plasmid DNA was extracted and purified using a QIAprep Spin Miniprep Kit purchased from Qiagen and following the standard protocols provided. Purified plasmids were sequenced by Eurofins Genomics using their sequencing service. Plasmid purities and concentrations were obtained using a NanoDrop ND-1000 spectrophotometer.

Forward and reverse primers were designed to contain a single point mutation (Phe109Tyr and Lys122Arg/Met/Glu/Gln). Primers were designed as follows:

Phe109Tyr Forward: 5’-CACTTCTATTTCTTCTGCACTTTCCCGGGTCAC-3’

Phe109Tyr Reverse: 5’-GCAGAAGAAATAGAAGTGTTCACCTTCTTTAAGCTTGG-3’

Lys122Arg Forward: 5’-CACTGATGCGTGGTACCCTGACTCTGAAATAG-3’

Lys122Arg Reverse: 5’-GTACCACGCATCAGTGCGGAGTGACC-3’

Lys122Met Forward: 5’-CACTGATGATGGGTACCCTGACTCTGAAATAG-3’

Lys122Met Reverse: 5’-GTACCCATCATCAGTGCGGAGTGACC-3’

Lys122Glu Forward: 5’-ACTGATGGAAGGTACCCTGACTCTGAAATAG-3’

Lys122Glu Reverse: 5’-GTACCTTCCATCAGTGCGGAGTGACC-3’

Lys122Gln Forward: 5’-CTGATGCAAGGTACCCTGACTCTGAAATAGAGATCC-3’

Lys122Gln Reverse: 5’-GTACCTTGCATCAGTGCGGAGTGACC-3’

3. Protein Expression, Purification, and Modification

Plasmids were transformed into competent BL21(DE3) *E. coli* cells obtained from NEB and selected using ampicillin-containing agar plates. Colonies were grown overnight at 37 °C and single colonies selected for liquid starter cultures in LB broth containing 100 µg/mL of ampicillin. Starter cultures were grown at 37 °C with 180 rpm of shaking for 6 hours. Overnight 1 L expression cultures were prepared by adding a small portion of starter culture (~ 3mL) into LB broth containing 100 µg/mL of ampicillin and 0.4% glycerol. Overnight expression cultures were grown at 37 °C with 180 rpm of shaking. Cells were then pelleted by centrifugation at 4000 rpm using a Beckman Avanti J-26XP centrifuge in a JLA 10.5000 rotor for 20 minutes for 20 minutes. Cell pellets were resuspended in osmotic shock buffer (50 mM Tris, 1 mM EDTA, 20% sucrose, pH 8.1) and rest on ice for 20 minutes. Cell pellets were then obtained by centrifugation at 7000 rpm using a Beckman Avanti J-26XP centrifuge in a JA 25.50 rotor for 20 minutes. Pellets were resuspended in 500 µM MgCl2 and rest on ice for 20 minutes. Proteins were isolated by centrifugation at 12000 rpm using a Beckman Avanti J-26XP centrifuge in a JA 25.50 rotor for 20 minutes. Supernatants were collected and 100 mM CuSO4 and 500 mM NaOAc (pH 4.5) were added dropwise. These mixtures were incubated overnight at 37 °C to promote Cu(II) uptake by apo-azurin. Acid precipitated proteins were removed by centrifugation at 12000 rpm using a Beckman Avanti J-26XP centrifuge in a JA 25.50 rotor for 20 minutes. The resulting supernatants were collected and purified on a CM Sepharose column using a NaOAc concentration gradient. Protein purities and concentrations were determined spectrophotometrically using reported extinction coefficients (ε(628 nm) = 5600 $^{-1}$ cm^{-1}).[2,3]

Labeled azurins were obtained by adding 1.1 equivalents of ($[Ru(bpy)_2CO_3]^{2+}$) to a solution of azurin in 300 mM $NaHCO_3$ (pH 7.5). These reaction mixtures were incubated overnight in the dark at room temperature with gentle agitation. FPLC was carried out using a HiTrap IMAC FF column that was obtained from GE Healthcare (now called Cytiva Life Sciences) and manually metalated with Cu^{2+}. Reaction mixture was exchanged into binding buffer (1 M NaCl, 10 mM Tris, pH 8) and loaded to the previously equilibrated column under the same buffer system. Unmodified azurins bound to the column while ruthenium-modified azurins (Ru-His107Tyr109Arg122, Ru-His107Tyr109Met122, Ru-His107Tyr109Glu122, and Ru-His107Tyr109Gln122) elute with the binding buffer wash. Unlabeled azurins were eluted using elution buffer (1 M NH4Cl, 20 mM NaPi, pH 7.8). Fractions were collected and monitored by UV-visible spectrophotometry to pool correct fractions together. Concentrations of each Ru-modified azurin was calculated spectrophotometrically using reported extinction coefficients (ε(628 nm) = 5,600 M^{-1} cm^{-1} for Cu(II) and ε(500 nm) = 10,000 M^{-1} cm^{-1} for Ru(II)).[4] Both proteins were exchanged into storage buffer (500 mM imidazole, 1 mM $CuSO_4$, 100 mM NaCl, pH 7.5) and incubated over the weekend. Long term storage is carried out in this buffer.

4. Tyrosine Fluorescence

Fluorescence experiments were carried out using a Horiba Jobin-Yvon Fluorolog. 65 µM samples were prepared in 50 mM NaPi at pH values from 4 to 11. Samples were placed into air-free cuvettes and deoxygenated with 15-20 pump-backfill cycles using N_2. Excitation was achieved using 280 nm light and spectra were recorded from 295 to 500 nm to monitor for Tyr and/or TyrO^{-} fluorescence. Ten scans were performed per sample and emission maxima were determined by averaging each series.

5. Electrochemistry

Electrochemical experiments were carried out using a CH Instruments 6171B potentiostat. A standard three-electrode setup was used containing a basal-plane graphite (BPG) working electrode, and platinum wire counter electrode, and an AgCl/Ag reference electrode with all samples being reported versus NHE. Protein samples (100 µM) were prepared in 100 mM NaOAc at pH values ranging from 5 to 9. The BPG electrode surface were prepared by thoroughly washing with deionized H2O, lightly coating with MicroPolish Powder (CH Instruments), and polishing using an microfibre polishing pad. CV spectra were collected using a 0.02, 0.04, and 0.1 V/s scan rate, however, the 0.02 V/s data are the only scans shown here unless otherwise noted. The scan window for DPV was -0.1 to 1 V at 0.003 V increments with a 0.05 V amplitude. The pulse and sample width were 0.1 and 0.02 s, respectively. The pulse period was 0.5 s.

6. Transient Absorption (TA) Spectroscopy

All laser experiments were carried out with a 480 nm excitation with 6 mJ/pulse energy. Samples were reduced using L-ascorbic acid and then desalted into 250 mM imidazole, 100 mM NaCl at various pH values (5-9) for a final concentration of 35 µM. Sample volumes were 1.5 mL and special air-free cuvettes were custom made by the SFU Glass Shop to allow for deoxygenation. 15-20 pump-backfill cycles using N_2 were performed to remove O_2 from the sample and cuvette. Prior to TA experiments, time-dependent fluorescence scans were performed, monitoring at 670 nm. The quencher $[Ru(NH_3)_6]Cl_3$, dissolved in 250 mM imidazole, 100 mM NaCl at the required pH, was added to a final concentration of 10 mM to each sample as an exogenous quencher. A total of 15-20 pump-backfill cycles using N_2 were performed again to remove O_2 from the sample and cuvette. Fluorescence experiments were repeated to ensure fluorescence quenching. TA was then carried out, monitoring at 490 nm pr 628 nm for Ru(II) reduction and Cu(I) oxidation, respectively. Data were plotted and analyzed using MATLAB and MATLAB's Curve Fitting Toolbox. Rate constants were determined using a two-exponential fit. The first exponential function describes signal from residual fluorescence due to excited state decay (and quenching) of the Ru photosensitizer and the second function describes the Cu(I) oxidation event. For the 490 nm (Ru traces), the first function is for excited state decay of *Ru(II) and the second function is for Ru(III).

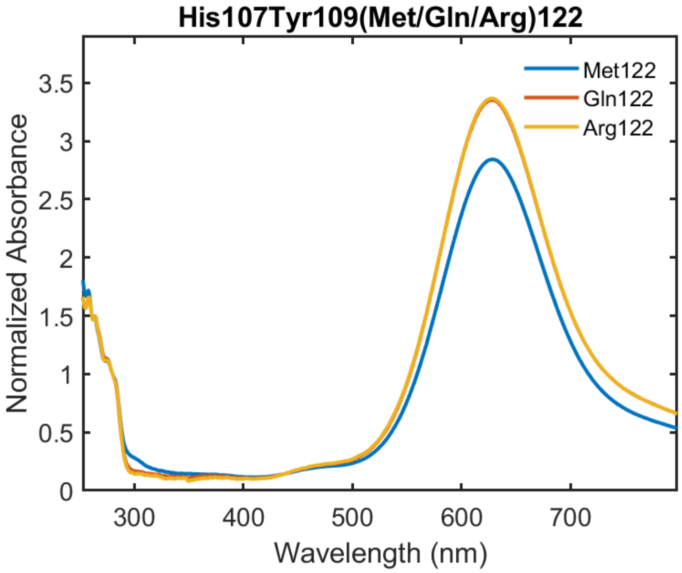

Figure S1. UV-visible spectra of His107Tyr109Met122 (blue), His107Tyr109Gln122 (orange), and His107Tyr109Arg122 (yellow). Absorbance was normalized to the 280 nm peak.

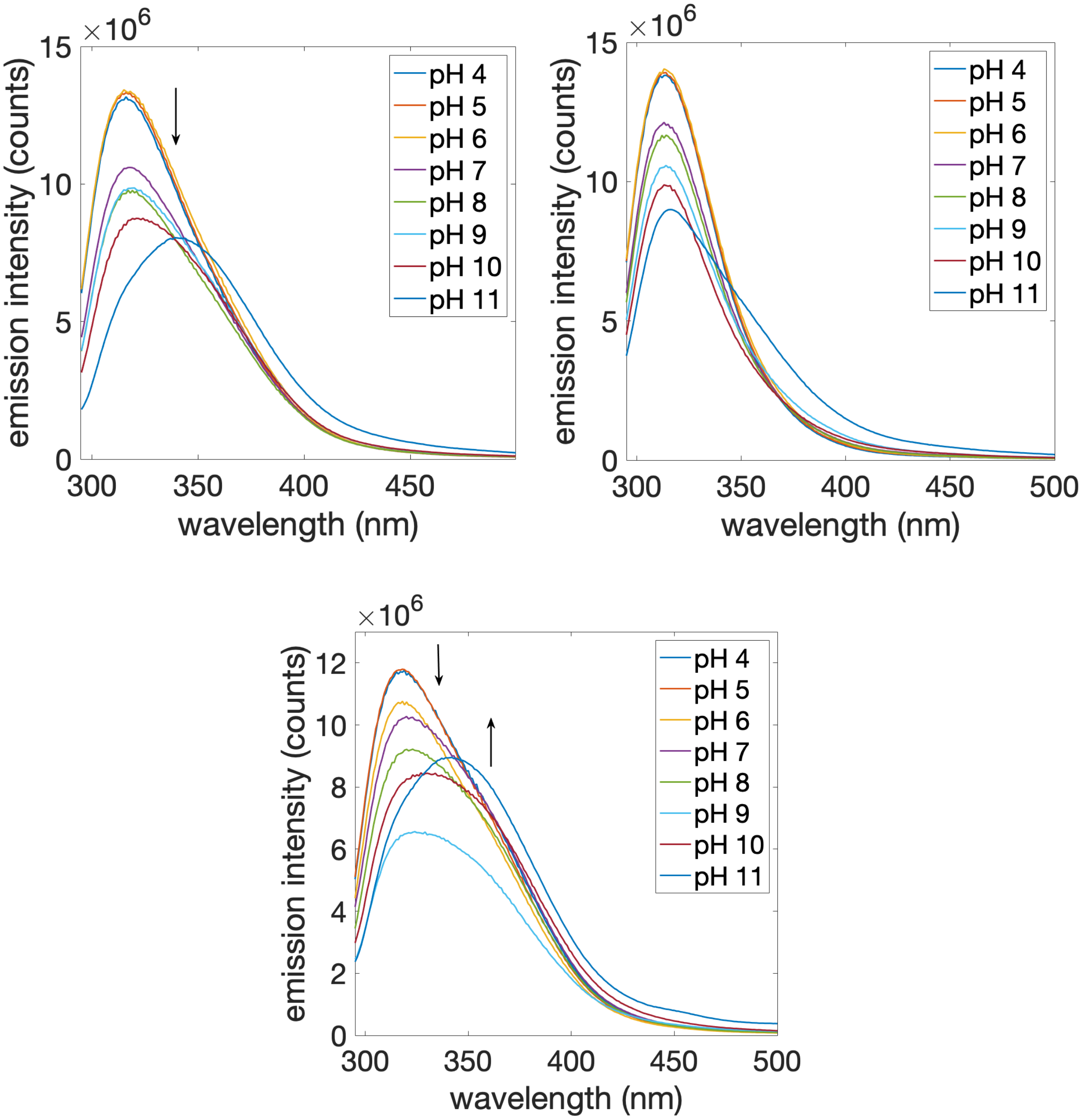

Figure S2. Fluorescence spectra of 60 μM solutions of His107Tyr109(Met/Gln/Arg)122 azurins in 50 mM sodium phosphate buffers. The excitation wavelength was 280 nm. All samples were deoxygenated and data collected under a N_2 atmosphere. Top left: Gln122. Top right: Met122. Bottom: Arg122

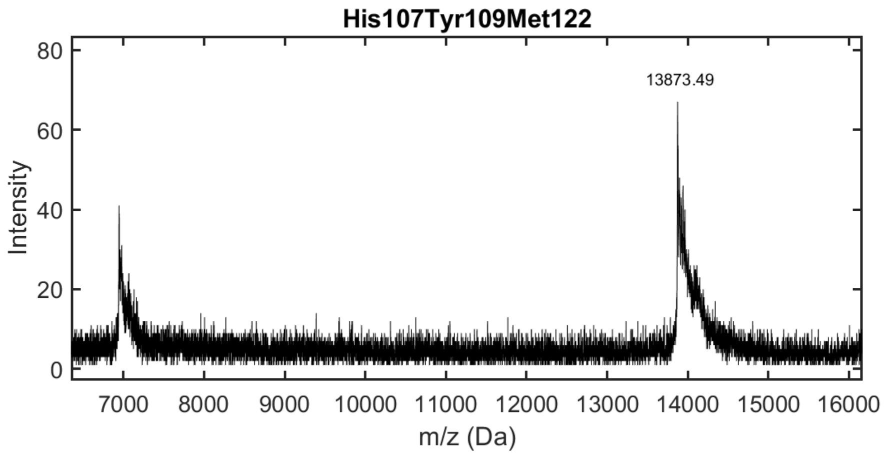

Figure S3. MALDI-TOF mass spectrometry of His107Tyr109Met122. Calculated mass: 13868.74 Da. Observed mass: 13873.49 Da.

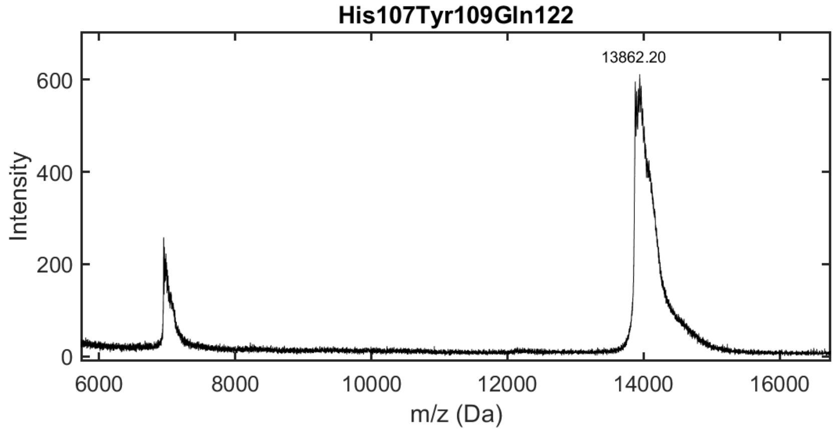

Figure S4. MALDI-TOF mass spectrometry of His107Tyr109Gln122. Calculated mass: 13865.68 Da. Observed mass: 13862.20 Da.

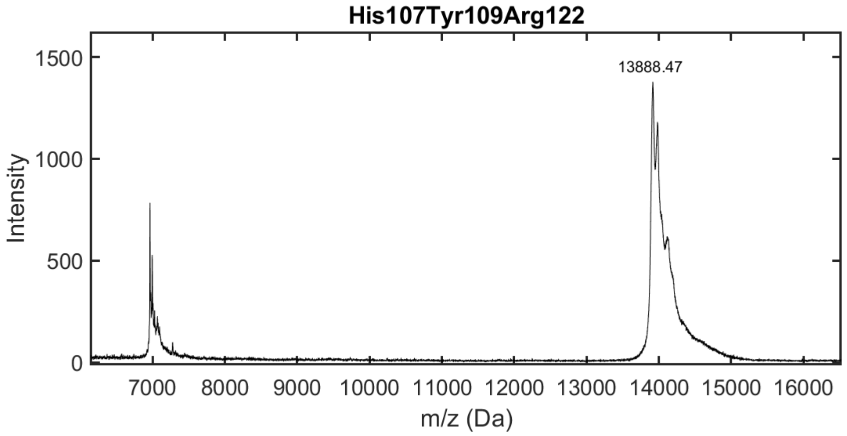

Figure S5. MALDI-TOF mass spectrometry of His107Ty109Arg122. Calculated mass: 13893.74 Da. Observed mass: 13899.47 Da.

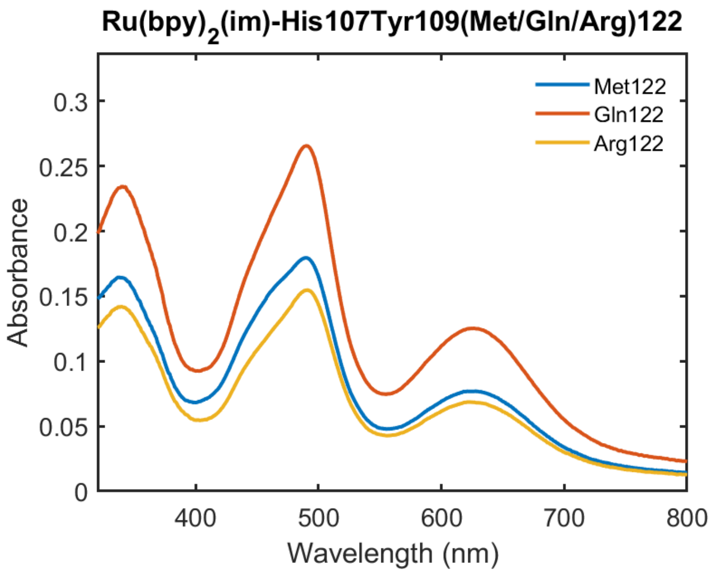

Figure S6. UV-vis spectra of Ru(2,2´-biyridyl)$_2$(imidazole)-labeled His107Tyr109Met122 (blue), His107Tyr109Gln122 (orange), and His107Tyr109Arg122 (yellow). Absorbance was not normalized and concentrations are different for each protein sample.

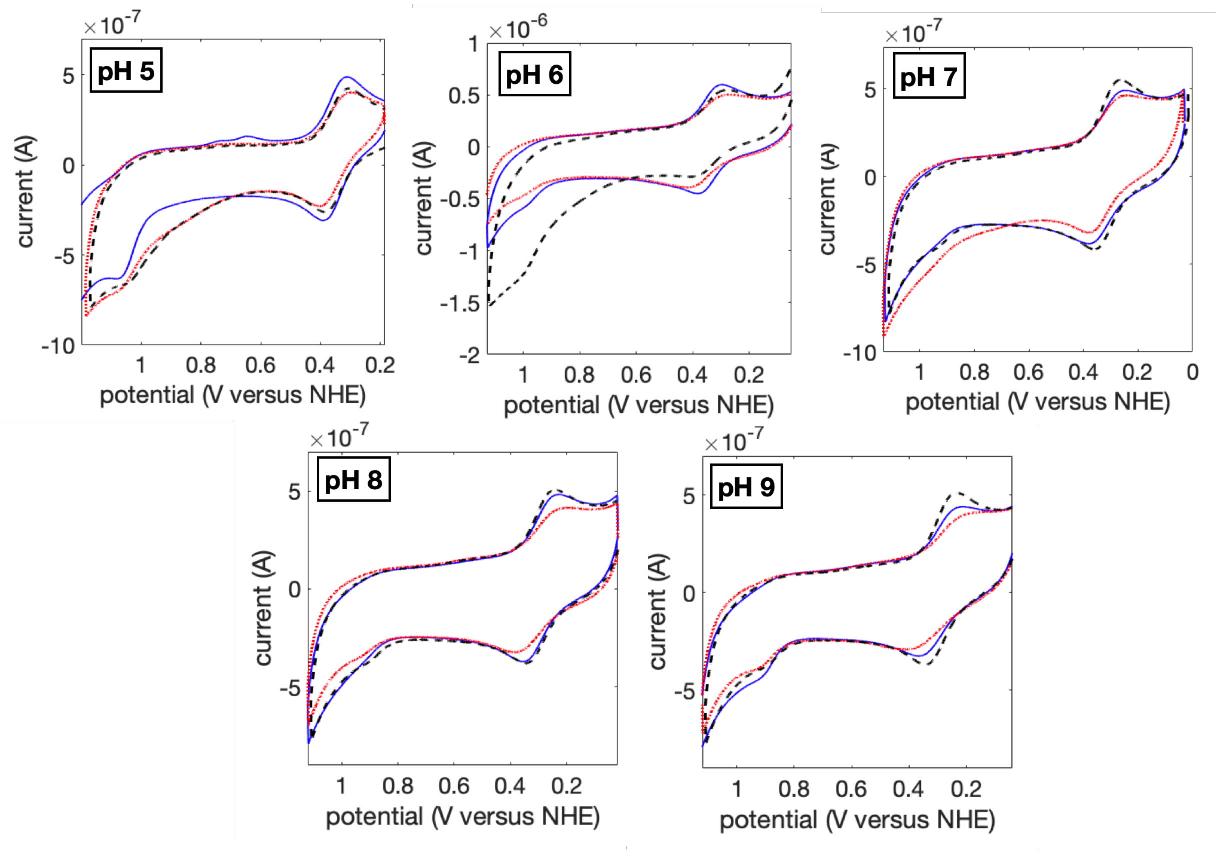

Figure S7. Cyclic voltammograms of 60 µM His107Tyr109(Met/Gln/Arg)122 azurins at pH 5-9 in 100 mM NaOAc solutions. The blue solid line shows the Met122 variant, the red dotted line shows the Gln122 variant, and the black dashed line shows the Arg122 variant.

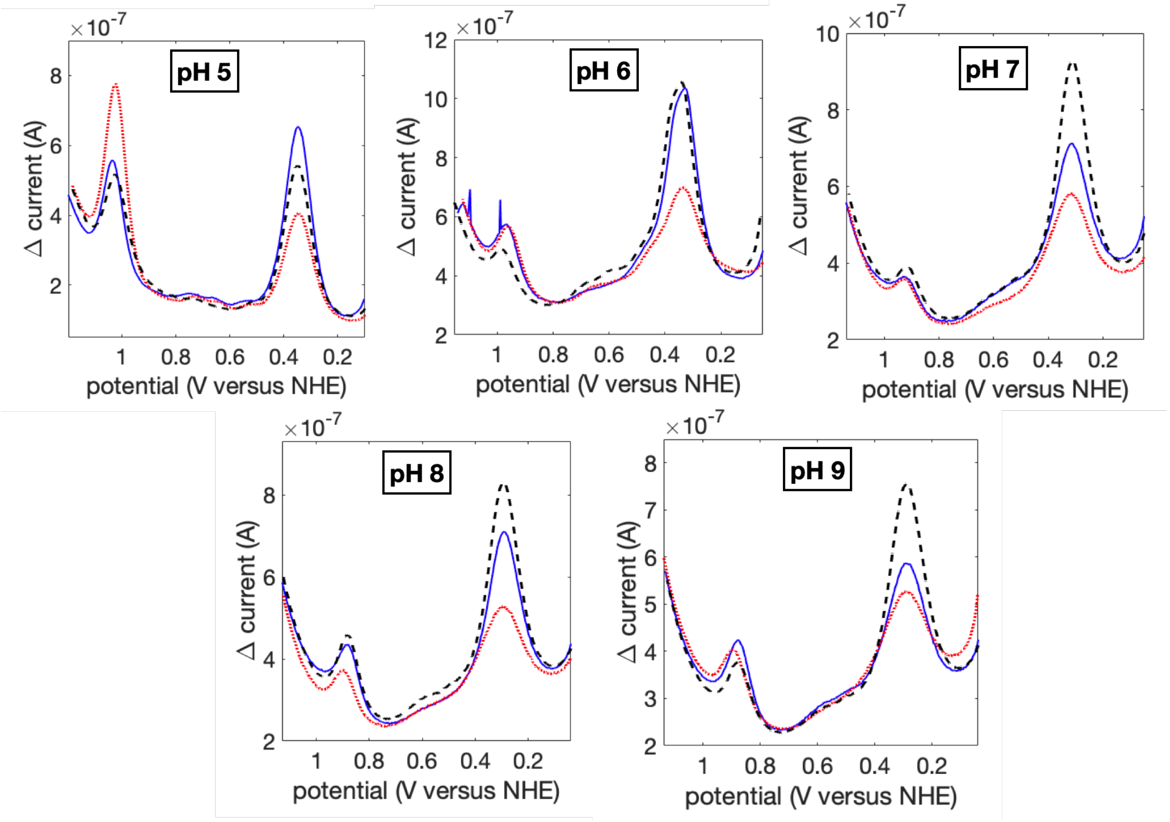

Figure S8. Differential pulse voltammograms of 60 µM His107Tyr109(Met/Gln/Arg)122 azurins at pH 5-9 in 100 mM NaOAc solutions. The blue solid line shows the Met122 variant, the red dotted line shows the Gln122 variant, and the black dashed line shows the Arg122 variant.

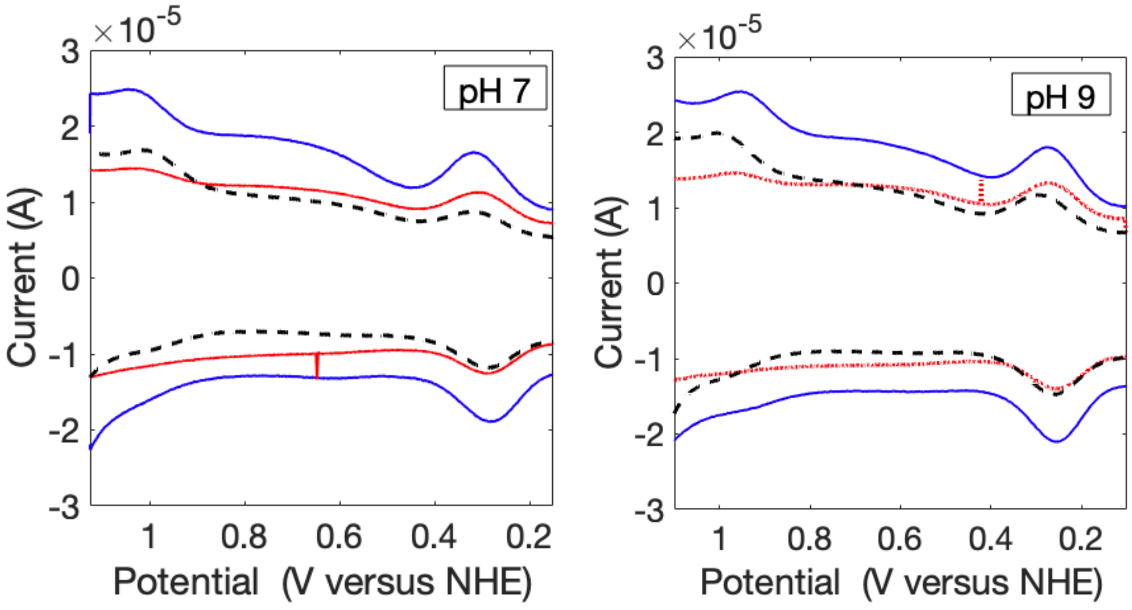

Figure S9. Examples of square wave voltammograms of 60 μM His107Tyr109(Met/Gln/Arg)122 azurins at pH 7 and 9 in 100 mM NaOAc solutions. The blue solid line shows the Met122 variant, the red dotted line shows the Gln122 variant, and the black dashed line shows the Arg122 variant.

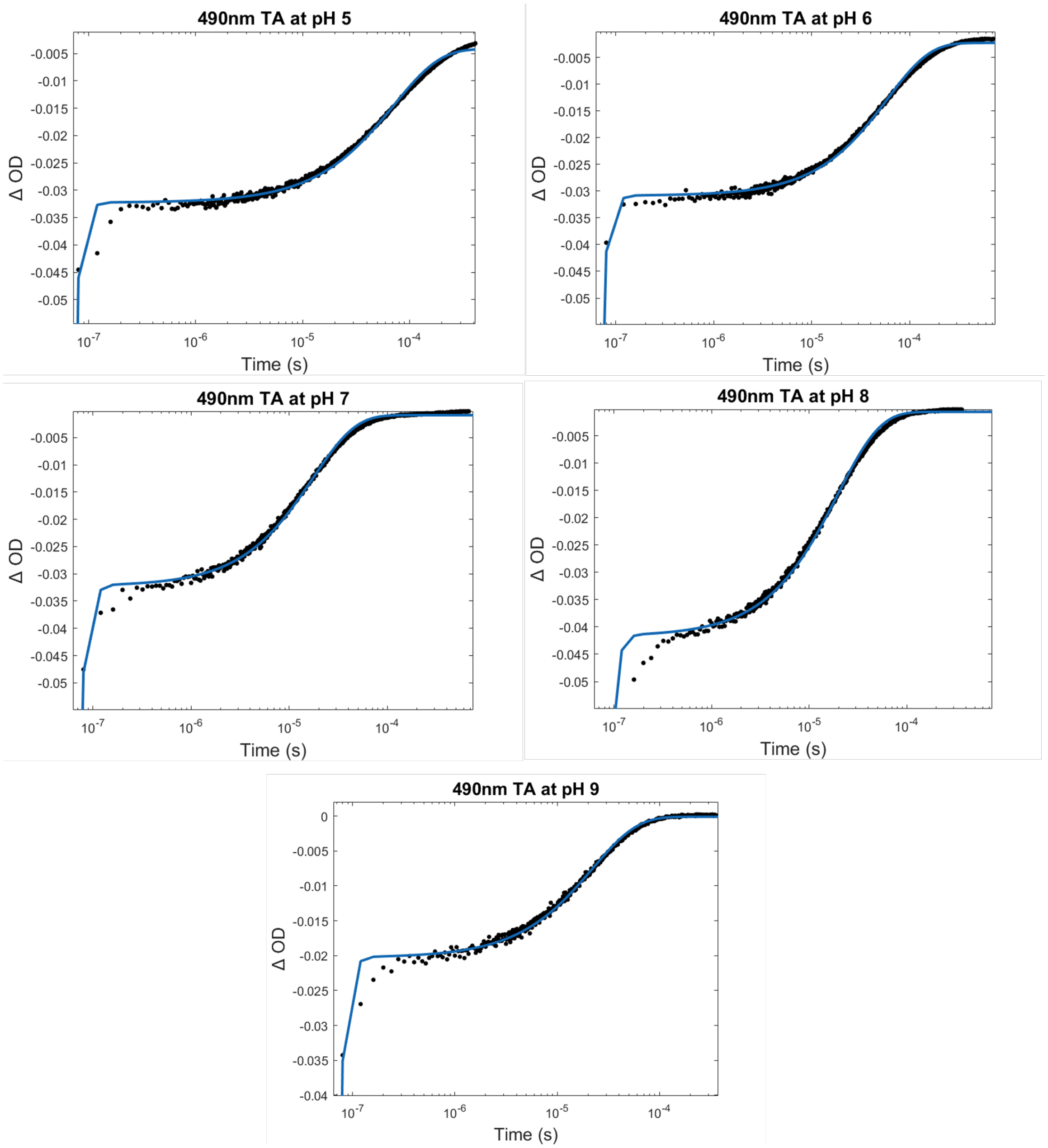

Figure S10. TA kinetic traces for the reduction of Ru(III) to Ru(II) in 35 μM His107Tyr109Met122 azurin across the pH range of 5-9. Experiments were carried out in 100 mM NaCl, 250 mM imidazole, at their respective pH values. $[Ru(NH_3)_6]Cl_3$ was added to a final concentration of 10 mM.

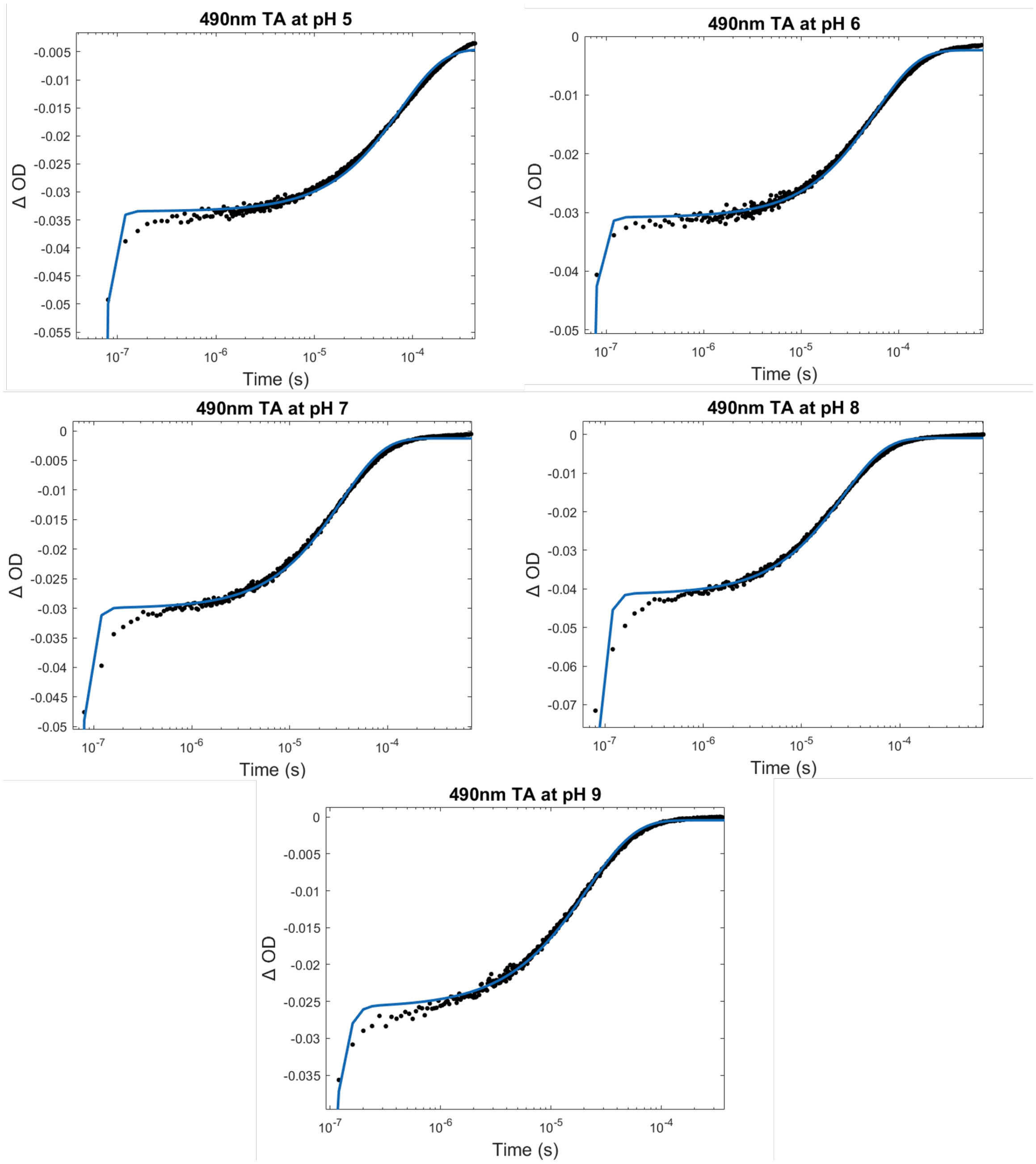

Figure S11. TA kinetic traces for the reduction of Ru(III) to Ru(II) in 35 μM His107Tyr109Gln122 azurin across the pH range of 5-9. Experiments were carried out in 100 mM NaCl, 250 mM imidazole, at their respective pH values. $[Ru(NH_3)_6]Cl_3$was added to a final concentration of 10 mM.

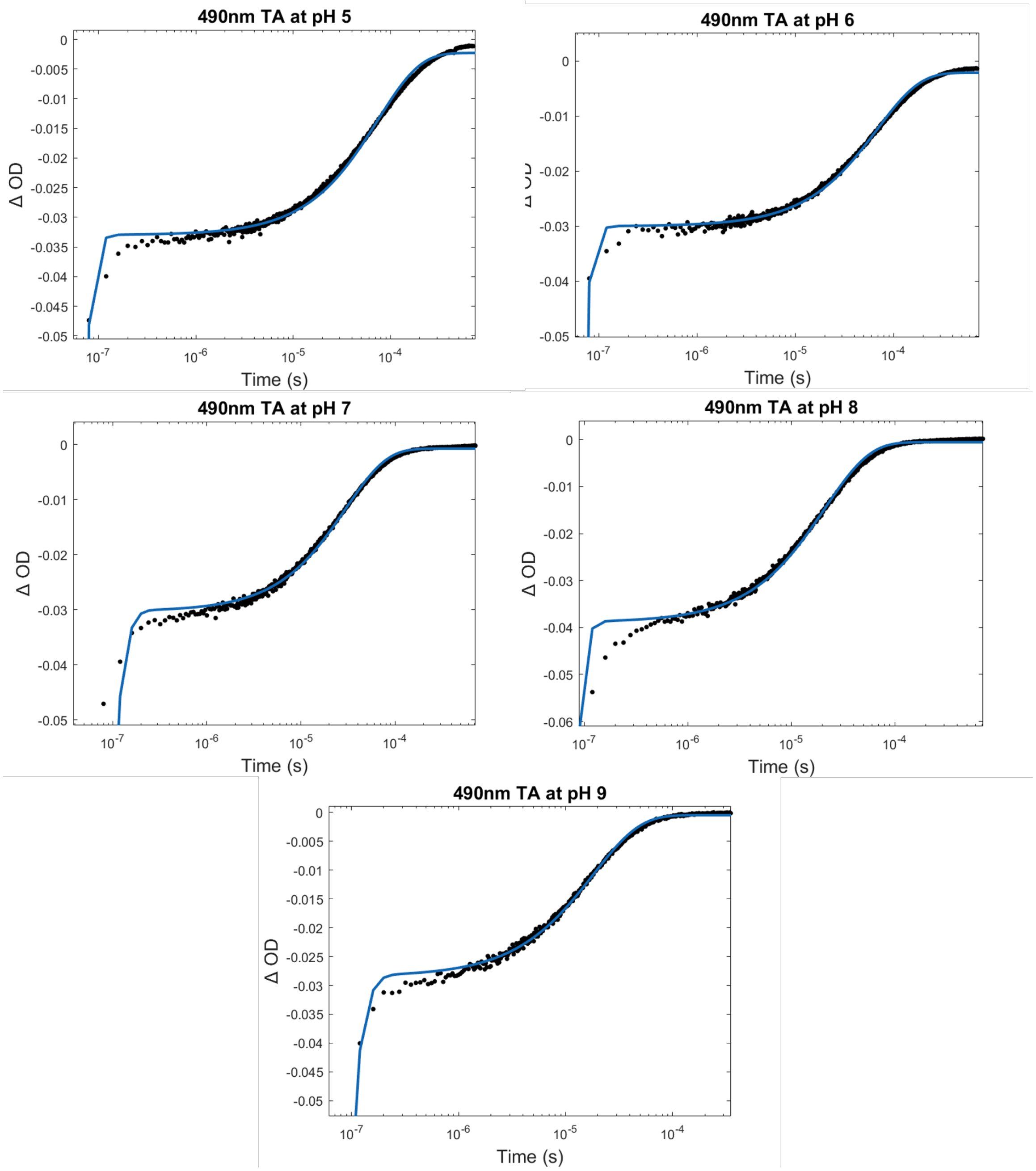

Figure S12. TA kinetic traces for the reduction of Ru(III) to Ru(II) in 35 μM His107Tyr109Arg122 azurin across the pH range of 5-9. Experiments were carried out in 100 mM NaCl, 250 mM imidazole, at their respective pH values. $[Ru(NH_3)_6]Cl_3$was added to a final concentration of 10 mM.

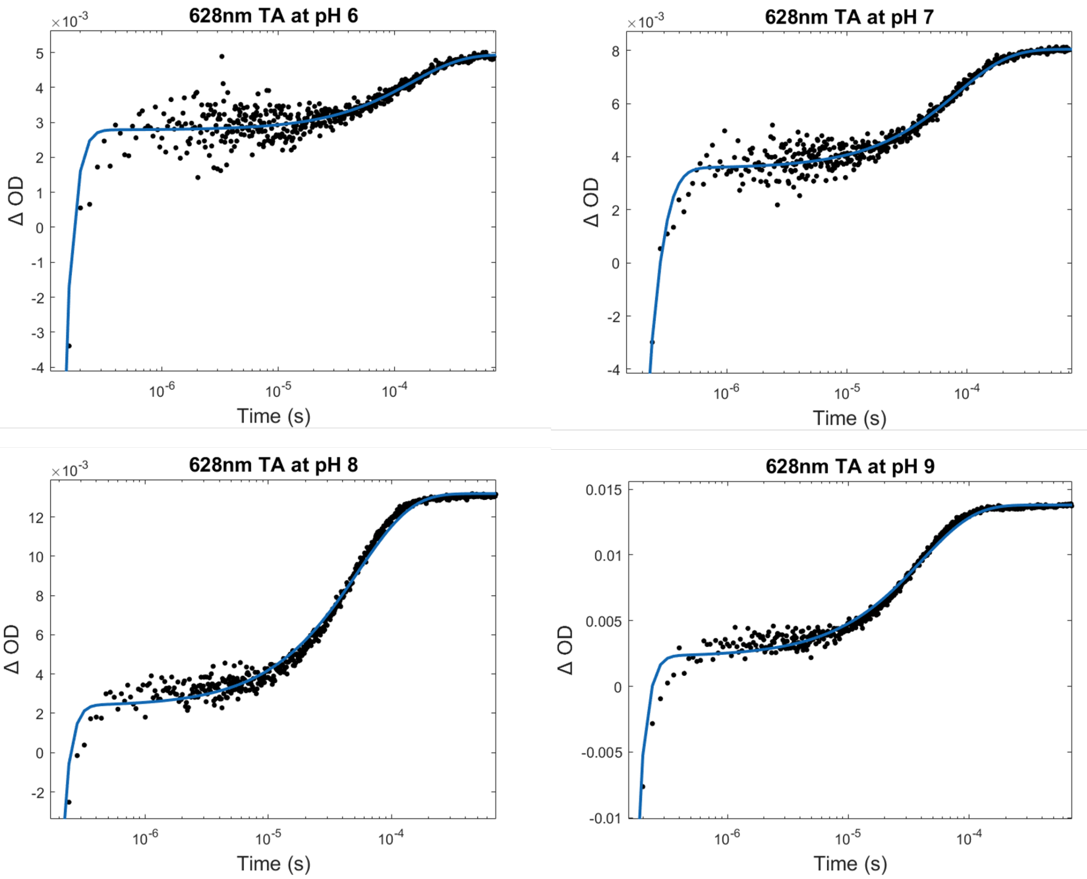

Figure S13. TA kinetic traces for the oxidation of Cu(I) to Cu(II) in 35 µM His107Tyr109Met122 azurin across the pH range of 5-9. Experiments were carried out in 100 mM NaCl, 250 mM imidazole, at their respective pH values. $[Ru(NH_3)_6]Cl_3$ was added to a final concentration of 10 mM.

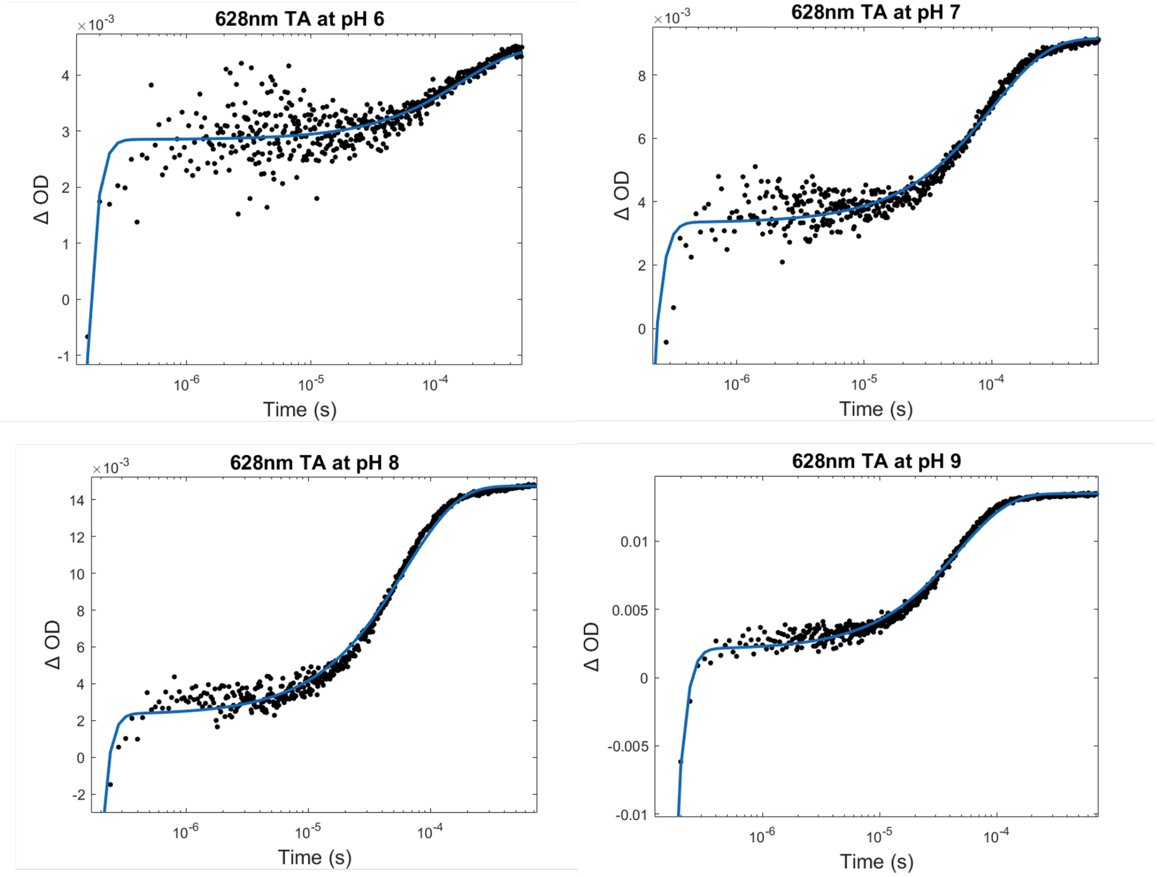

Figure S14. TA kinetic traces for the oxidation of Cu(I) to Cu(II) in 35 μM His107Tyr109Gln122 azurin across the pH range of 5-9. Experiments were carried out in 100 mM NaCl, 250 mM imidazole, at their respective pH values. $[Ru(NH_3)_6]Cl_3$was added to a final concentration of 10 mM.

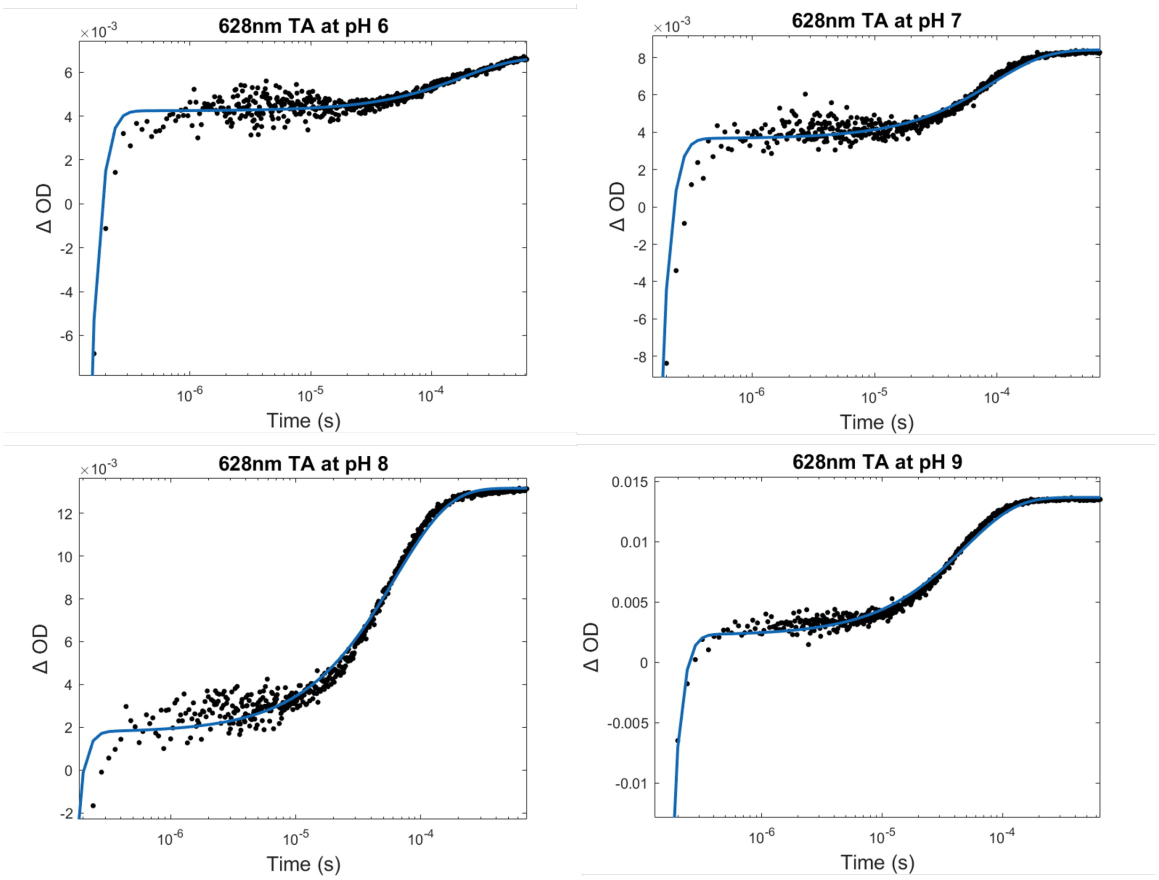

Figure S15. TA kinetic traces for the oxidation of Cu(I) to Cu(II) in 35 μM His107Tyr109Arg122 azurin across the pH range of 5-9. Experiments were carried out in 100 mM NaCl, 250 mM imidazole, at their respective pH values. $[Ru(NH_3)_6]Cl_3$ was added to a final concentration of 10 mM.

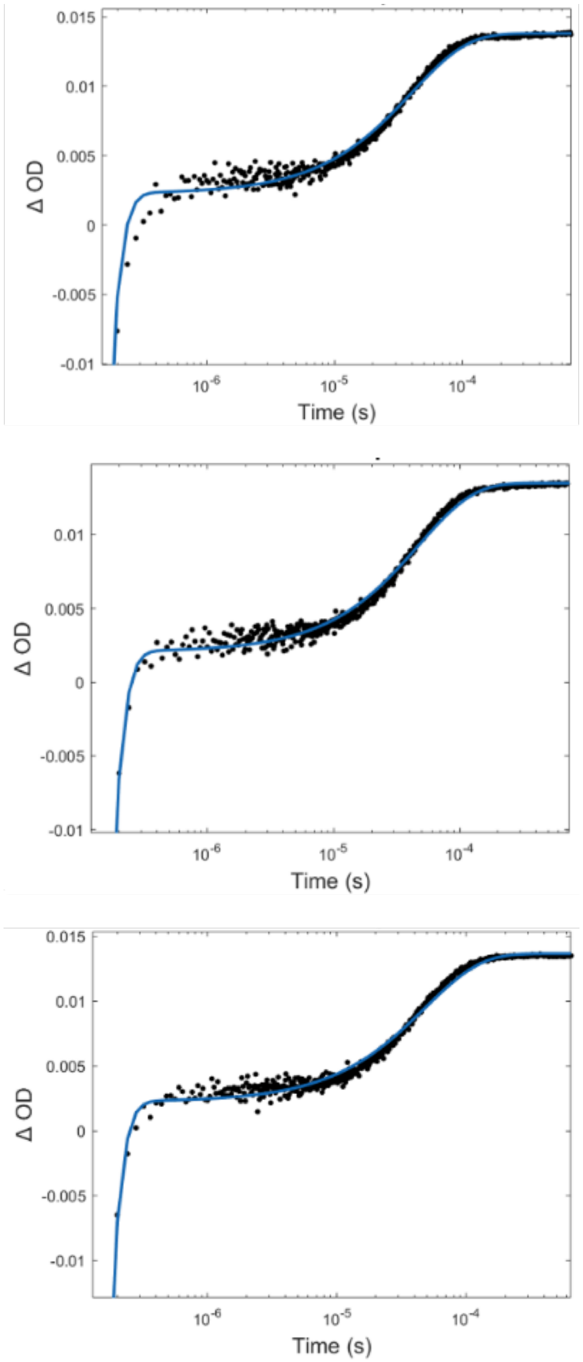

Figure S16. Comparison of TA kinetic traces for the oxidation of Cu(I) to Cu(II) in 35 μM His107Tyr109X122 azurins at pH 9, where the rate differences are largest. The top panel is where X= M122, the middle is for X = Q122, and the bottom is for X = R122. Experiments were carried out in 100 mM NaCl, 250 mM imidazole, at their respective pH values. $[Ru(NH_3)_6]Cl_3$ was added to a final concentration of 10 mM.

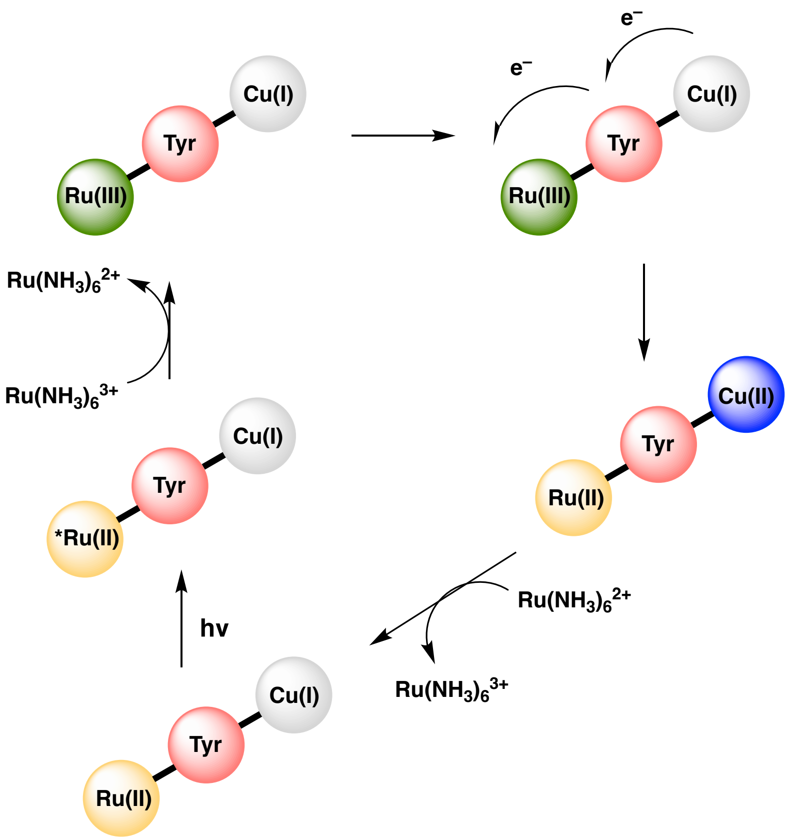

Figure S17. A general reaction scheme for investigation electron hopping using flash-quench generated Ru(III) oxidants. Starting from the bottom left panel

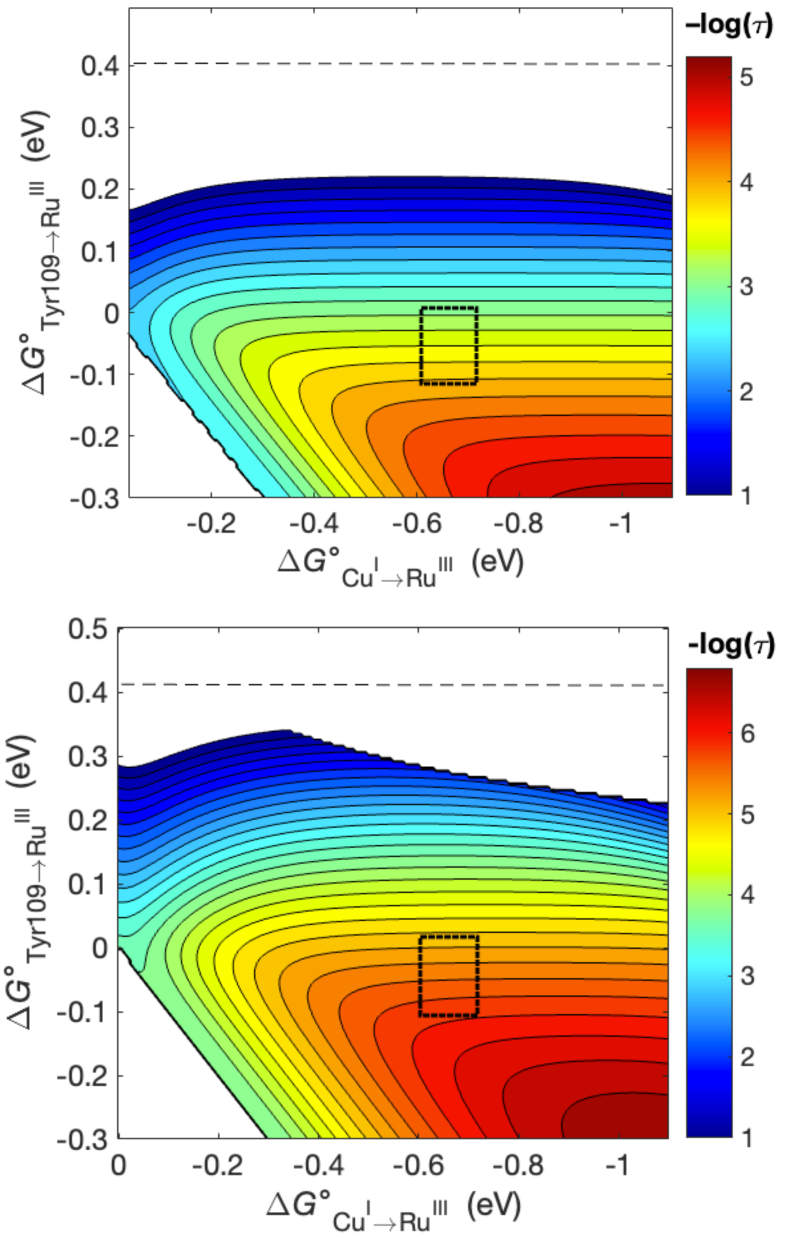

Figure S18. Comparison of hopping maps for RuHis107Tyr109X122 azurins. The top map was generated using through-bond distances where r(Ru-Tyr109) = 16.5 Å, r(Tyr109-Cu) = 18.3 Å, and r(Ru-Cu) = 34.8 Å. The bottom map was generated using through-space distances from the His 107 centroid to the Tyr109 centroid to the Cu ion where r(Ru-Tyr109) = 12.2 Å, r(Tyr109-Cu) = 15.7 Å, and r(Ru-Cu) = 25.8 Å. For both maps, the λ value was 0.8 eV, T = 298K, and the distance decay constant $\beta = 1.1$ Å^{-1}. The dashed line at the top shows the driving force for ET from Tyr109 to Ru^{III} assuming a potential of 1.4 V for Tyr109$^{\bullet+/0}$. The dashed rectangle indicates the driving force regime relevant here.

References.

[1] L. Zheng, U. Baumann, J.-L. Reymond, An Efficient One-Step Site-Directed and Site-Saturation Mutagenesis Protocol, Nucleic Acids Res. 32 (2004) e115–e115. https://doi.org/10.1093/nar/gnh110.
[2] B.P. Fedoretz-Maxwell, C.H. Shin, G.A. MacNeil, L.J. Worrall, R. Park, N.C.J. Strynadka, C.J. Walsby, J.J. Warren, The Impact of Second Coordination Sphere Methionine-Aromatic Interactions in Copper Proteins, Inorg. Chem. 61 (2022) 5563–5571. https://doi.org/10.1021/acs.inorgchem.2c00030.
[3] J. Liu, S. Chakraborty, P. Hosseinzadeh, Y. Yu, S. Tian, I. Petrik, A. Bhagi, Y. Lu, Metalloproteins Containing Cytochrome, Iron–Sulfur, or Copper Redox Centers, Chem. Rev. 114 (2014) 4366–4469. https://doi.org/10.1021/cr400479b.
[4] S. Faham, M.W. Day, W.B. Connick, B.R. Crane, A.J. Di Bilio, W.P. Schaefer, D.C. Rees, H.B. Gray, Structures of Ruthenium-Modified *Pseudomonas aeruginosa* Azurin and [Ru(2,2′-ipyridine)$_2$(imidazole)$_2$]$SO_4 \cdot 10H_2O$, Acta Crystallogr. D. 55 (1999) 379–385. https://doi.org/10.1107/S0907444998010464.
